# Spatial analysis of infection by the human immunodeficiency virus among pregnant women^1^

**DOI:** 10.1590/0104-1169.0481.2574

**Published:** 2015-07-03

**Authors:** Eliane Rolim de Holanda, Marli Teresinha Gimeniz Galvão, Nathália Lima Pedrosa, Simone de Sousa Paiva, Rosa Lívia Freitas de Almeida

**Affiliations:** 2 PhD, Adjunct Professor, Centro Acadêmico de Vitória, Universidade Federal de Pernambuco, Vitória de Santo Antão, PE, Brazil; 3 PhD, Associate Professor, Departamento de Enfermagem, Universidade Federal do Ceará, Fortaleza, CE, Brazil; 4 Master's student, Departamento de Enfermagem, Universidade Federal do Ceará, Fortaleza, CE, Brazil. Scholarship holder from Coordenação de Aperfeiçoamento de Pessoal de Nível Superior (CAPES), Brazil; 5 PhD, RN, Unidade Básica de Saúde, Secretaria Municipal de Saúde, Fortaleza, CE, Brazil; 6 Post-doctoral fellow, Departamento de Saúde Comunitária, Universidade Federal do Ceará, Fortaleza, CE, Brazil. Professor, Departamento de Saúde Comunitária, Universidade Federal do Ceará, Fortaleza, CE, Brazil. Scholarship holder from Coordenação de Aperfeiçoamento de Pessoal de Nível Superior (CAPES), Brazil

**Keywords:** Spatial Analysis, HIV, Health Status Indicators, Health Inequalities

## Abstract

**OBJECTIVES:**

to analyze the spatial distribution of reported cases of pregnant women infected by the human immunodeficiency virus and to identify the urban areas with greater social vulnerability to the infection among pregnant women.

**METHOD:**

ecological study, developed by means of spatial analysis techniques of area data. Secondary data were used from the Brazilian National Disease Notification System for the city of Recife, Pernambuco. Birth data were obtained from the Brazilian Information System on Live Births and socioeconomic data from the 2010 Demographic Census.

**RESULTS:**

the presence of spatial self-correlation was verified. Moran's Index was significant for the distribution. Clusters were identified, considered as high-risk areas, located in grouped neighborhoods, with equally high infection rates among pregnant women. A neighborhood located in the Northwest of the city was distinguished, considered in an epidemiological transition phase.

**CONCLUSION:**

precarious living conditions, as evidenced by the indicators illiteracy, absence of prenatal care and poverty, were relevant for the risk of vertical HIV transmission, converging to the grouping of cases among disadvantaged regions.

## Introduction

Current trends suggest a close relation between the social determination of Aids and the environment. This problem does not show a unique epidemiological profile and is distributed heterogeneously; it varies geographically among regions, states, cities and, in the case of big cities, among intra-urban spaces^1^^-^ 4. Therefore, it is essential to investigate all determinants, including social and economic determinants, attributed to the infection by the Human Immunodeficiency Virus (HIV).

Epidemiological data demonstrate the intensification of the feminization process of the epidemic, an aspect that exposes children to the disease through the vertical transmission of HIV. A study demonstrated that women's greater vulnerability to the infection derives from the individual dimension, as evidenced by the low education level, multiple sexual partners, history of Sexually Transmitted Diseases (STD), irregular condom use, as well as from the social and program dimensions, expressed by the epidemic^5^.

In that sense, the need for a deeper understanding of the spatial distribution of reported cases of HIV/Aids among pregnant women was evidenced, so as to get to know the socioterritorial dynamics of the infection in this population group and the possible risk areas that are considered most vulnerable. The assessment of the relations between illness and geographical space permits identifying specific demands, priority sites and more effective control interventions, with a view to reducing the morbidity and mortality due to this problem.

By integrating the occurrence of cases reported in the health information systems with relevant spatial issues, the study can support public managers in the incorporation of innovative program strategies and in the territory-based prevention of vertical HIV transmission. Working directly in the maternal-infant health area, Nursing engages in these aspects and should participate, together with the health team, in finding solutions to cope with the social determinants of health related to illness caused by HIV/Aids.

The objective in this research was to analyze the spatial distribution of reported cases of pregnant women infected by the human immunodeficiency virus and to identify the areas with the greatest social vulnerability for this infection among pregnant women.

## Method

An ecological study was developed through the application of spatial analysis techniques to area data. The analysis units were the neighborhoods of the city of Recife, the state capital of Pernambuco, in the Northeast of Brazil, with about 1,536,934 inhabitants. Its territory is divided in six regions, defined as the Center, North, Northwest, West, Southwest and South.

The population included all cases of pregnant women seropositive for HIV, reported in the Brazilian National Disease Notification System (SINAN) between January 1^st^ 2001 and December 31^st^ 2011, who lived in Recife. Duplicated registered were excluded and considered only once to avoid duplicated information. Pregnant women who evolved to abortion or who gave birth to a stillborn child were also excluded, aiming to exclusively analyze the potential risk of infection by vertical HIV transmission of live borns, similar to other studies that adopted this criterion^6^^-^ 8. The city's Epidemiological Surveillance Sector made available the databases used in this study.

In total, 1,614 notifications had been registered. These included 14 duplicated cases, 623 who did not live in Recife, 77 beyond the established time period, 48 who evolved to abortion and 11 cases of still born children, all of whom were excluded. Hence, 841 notifications were eligible for the study. Birth data were taken from the National information System on Live Births (SINASC). The socioeconomic data were obtained from the 2010 Demographic Census, per census sector, and then aggregated per neighborhood.

The cartographic base that contains the official neighborhoods of Recife was obtained from the Cartographic Engineering Department of the Universidade Federal de Pernambuco. The cartographic projection corresponded to the Mercator Universal Transversa system, using the horizontal model Terra Datum SAD 1969 and the zone 25S.

The HIV detection rate was calculated in pregnant women, per neighborhood, using the new cases in each neighborhood as the numerator and the population of pregnant women as the denominator, estimated by the number of live births in each neighborhood in the study period.

The Freeman-Tukey method was used to transform this rate, in order to correct the random fluctuations in small populations and identify spatial patterns based on rates mitigated by the reduced variance of their coefficients. Next, a thematic map was produced with the spatial distribution of the HIV detection rates in pregnant women, stratified in four classes, by means of the quantile method.

Inequalities in the city's socio-spatial profile were identified by calculating the socioeconomic indicators per neighborhood, graphically represented through thematic maps. The following indicators were chosen, as they reflect situations of social vulnerability: illiteracy rate, proportion of pregnant women without prenatal care, homes in the poverty range and illiterate women responsible for the home. In this phase, exploratory analysis was developed through a visual inspection of the constructed maps.

The variable "neighborhood" was combined with the cartographic base for the georeferencing of the data. In those cases in which the addresses were incomplete or contained ortographic errors, the databases were corrected through research in Google Earth, always considered the pregnant woman's address at the time of the notification. This location considered the total area of the polygon representing each neighborhood.

To determine whether a spatially conditioned pattern existed in the detection rates of HIV in pregnant women, the Moran Index was used, identifying area clusters with similar risks for the occurrence of the outcome of interest. This index is able to detect the existence of spatial patterns, that is, the similarity among areas^9^. A neighborhood matrix was constructed, obtained through the contiguity criterion. Significance was set at 5%.

Then, Moran's scattering diagram was used to compare the score of each neighborhood studied with its neighbors. The areas located in quadrants Q1(high-high) and Q2 (low-low), visually represented using BoxMAP, indicate a positive spatial self-correlation, that is, neighborhoods with scores similar to that of their neighbors in terms of the HIV infection rates, characterizing spatial clusters. Quadrants Q3 (high-low) and Q4 (low-high), in turn, represent a negative spatial self-correlation, that is, neighborhoods with infection rates that are distant from their neighbors, thus characterizing discrepant observations^10^. This shows the distribution of the spatial patterns, identifying a trend towards homogeneous clusters (Q1 and Q2) and areas in transition (Q3 and Q4), regarding the data of the pregnant women with HIV in the urban areas of the city.

In the next phase, the Local Indicator of Spatial Association (LISA) was applied to detect regions with a local spatial correlation that significantly differs from the other data (p < 0.05%). The local indicators produce a specific coefficient for each area, favoring the identification of clusters, as seen on the LisaMap. The local indicators are classified as non-significant and significant at 95%, 99% and 99.9%^10^.

The final phase of the analysis mixed the zones with a positive spatial relation, as identified by the BoxMap, with spatial statistical significance superior to 95%, identified by the LisaMap, producing the MoranMap, which is useful to visualize the clusters and indicate priority areas. Critical areas were considered as neighborhoods in class Q1 (high-high) of the MoranMap^10^. The MoranMap only shows areas with significant LISA coefficients (p < 0.05), but classified in four groups, according to the quadrants they belong to in the scattering graph. The remaining areas, without spatial dependence, are classified as "not significant". Hence, the analysis units in the study were the neighborhoods with statistical significance on the MoranMap, as critical areas for the occurrence of HIV among pregnant women.

To process the data and construct the rates, the statistical software Statistical Package for Social Sciences (SPSS), 20.0 was used. The thematic maps, spatial self-correlation calculations and significance tests were obtained using resources from the software Terraview v4.2.2 and the spatial modules of the software R v2.15.3.

Approval for the study was received from the Institutional Review Board of the Health Sciences Center at Universidade Federal de Pernambuco, registered under protocol 645.546.

## Results

Between 2001 and 2011, the mean HIV detection rate in pregnant women living in Recife corresponded to 3.55 cases/1,000 live births.

**Figure 1.**
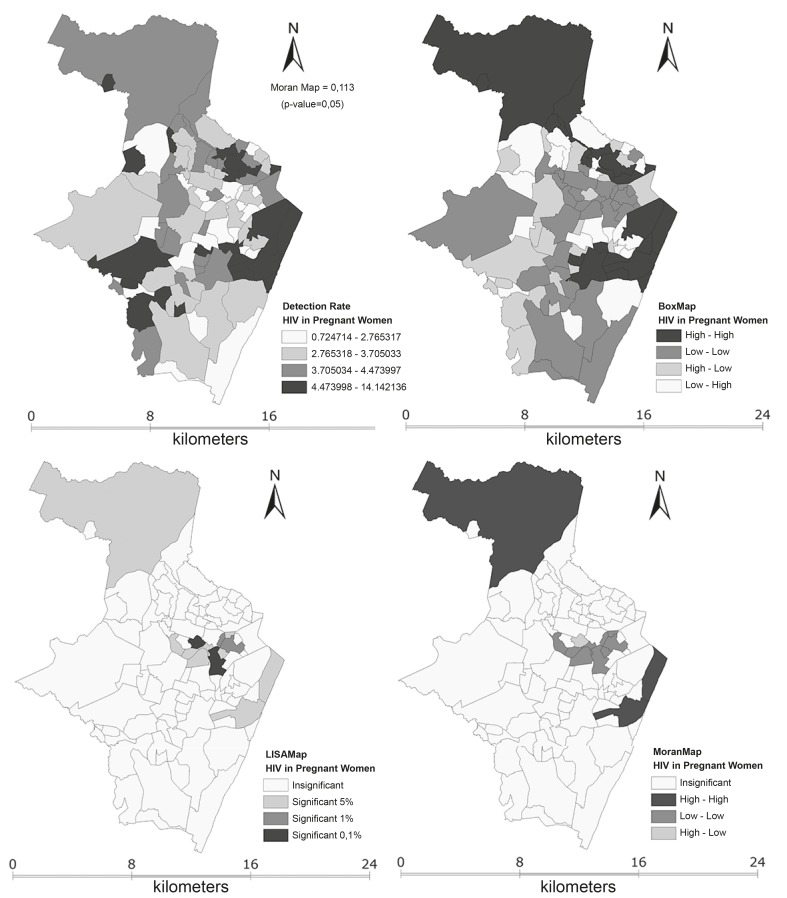
Spatial analysis of HIV detection rate in pregnant women between 2001 and 2011, Recife, PE, Brazil. A) Spatial distribution with Moran Index; B) BoxMap; C) LisaMap; D)t

Figure 1 illustrates the spatial analysis of the HIV detection rate in pregnant women during the research period. The spatial distribution of this rate by neighborhood revealed a concentration of higher coefficients in the Center and part of the Southwest, North and Northwest of the city (Figure 1A). The presence of spatial self-correlation was verified. The Moran Index was significant for this distribution (I=0.113; p-value=0.05), indicating the existence of clusters. Based on the positive value of the Moran test, it can be inferred that neighboring areas have similar HIV detection rates in pregnant women. Hence, neighborhoods with high coefficients of seropositive pregnant women are located near others with the same profile, while neighborhoods with low coefficients are near other neighborhoods with the same characteristic.

The BoxMap of Figure 1B illustrates areas classified according to their position on the Moran scattering diagram. Clusters with high rates of seropositive pregnant women are perceived, as well as neighbors with similar rates (high-high), mainly concentrated in the Center (Recife, São José, Brasília Teimosa, Soledade, Santo Amaro, Santo Antônio, Ilha Joana Bezerra, Cabanga), Southwest (Afogados, Mustardinha, Ilha do Retiro), part of the North (Linha do Tiro, Bomba do Hemetério, Água Fria, Arruda, Peixinhos) and Northwest (Alto José Bonifácio, Guabiraba, Pau-Ferro, Passarinho, Brejo da Guabiraba, Brejo de Beberibe). In addition, clusters with low rates of seropositive pregnant women and neighbors with similar coefficients (low-low) are located in the South (Boa Viagem, Imbiribeira, Jordão, Ibura), Southwest (Areias, Jiquiá, Jardim São Paulo, San Martin), North (Torreão, Encruzilhada, Rosarinho, Hipódromo, Cajueiro), Northwest (Santana, Casa Forte, Poço, Jaqueira, Tamarineira, Casa Amarela, Morro da Conceição, Monteiro, Alto do Mandu, Apipucos, Graças, Espinheiro, Aflitos) and part of the West (Cordeiro, Várzea, Torre).

Areas classified as going through epidemiological transition are revealed^9^, showing neighborhoods with opposite rates, as represented by high and low HIV detection rates in pregnant women. One continuing area is predominant, which goes from the South to the Northwest of the city, including neighborhoods with high HIV detection rates, but without clusters, as their neighbors show low coefficients (high-low). In addition, low rates were detected amidst neighborhoods with high rates (low-high), located is areas scattered around the city. These are exceptions with low coefficients without clusters, amidst high coefficients (Figure 1B).

In Figure 1C, the LisaMap evidenced clusters of neighborhoods that stand out from the others, with statistically significant local spatial dependence, at 0.1%, 1% and 5%. These are located in the Center, Northwest and North of the city.

The results found on the MoranMap are displayed in Figure 1D. This figure shows a cluster that is considered a high-risk area for HIV infection in pregnant women, due to the grouping of neighborhoods with high rates (high-high). This area includes the neighborhoods Recife and São José, located in the center of the city, as well as Guabiraba, in the Northwest. Another cluster is shown with low rates (low-low), punctually concentrated in the Northers (Rosarinho, Encruzilhada, Ponto de Parada), Western (Torre) and Northwestern (Graças, Aflitos, Jaqueira, Santana, Poço) adjacencies of the city. These neighborhoods represent low-risk areas for vertical HIV transmission.

The neighborhood Parnamirim is highlighted, located in the Northwest of the city, which is considered a transition areas, as it contains opposite rates, that is, high rates, but with neighboring areas showing low rates (high-low). The neighborhoods that are considered insignificant do not fit into the cluster, as they show varying HIV detection rates, as well as the rates in the neighboring areas (Figure 1D).

**Figure 2.**
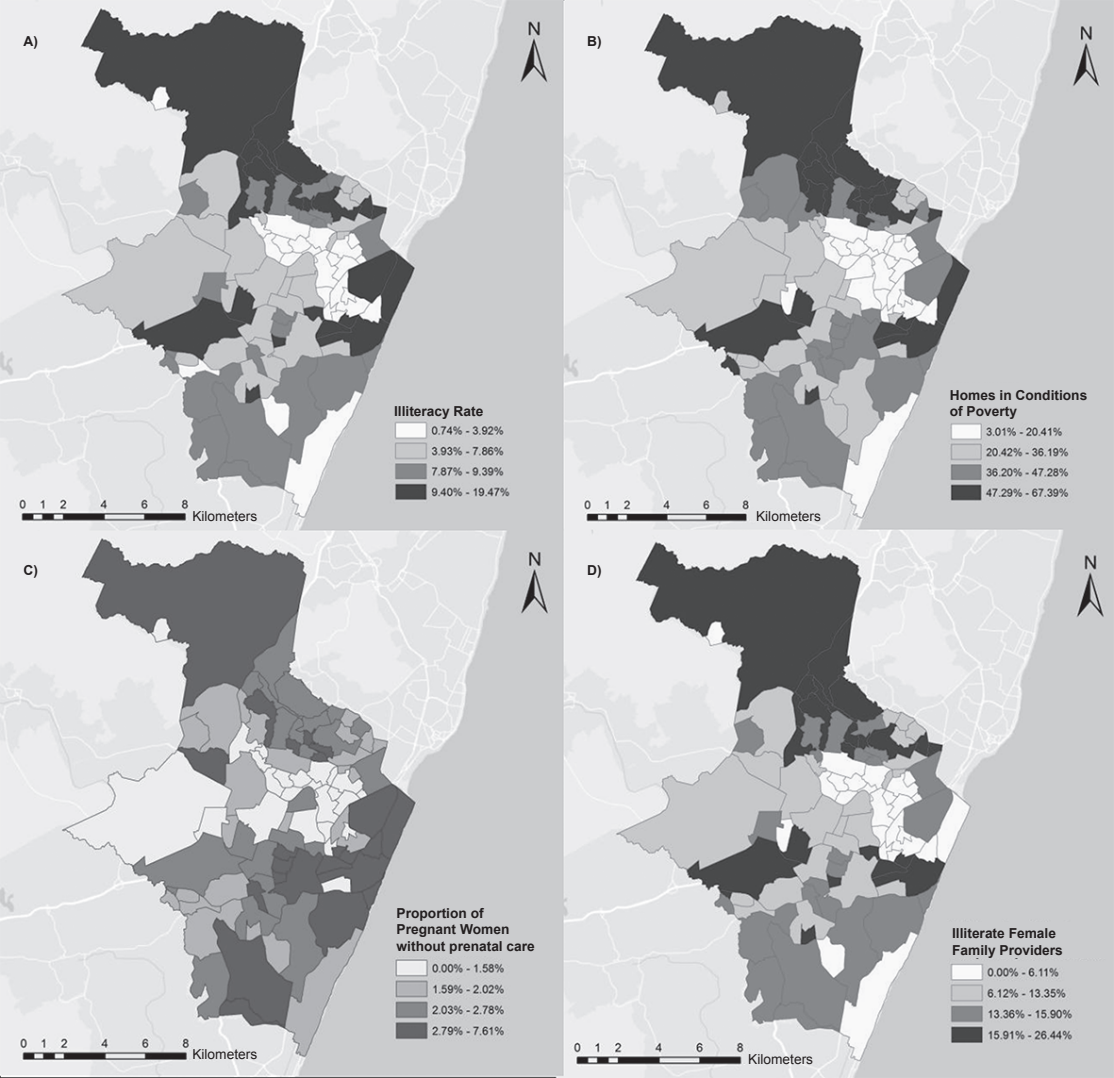
Spatial distribution of illiteracy rate, homes in poverty conditions, proportion of pregnant women without prenatal care and illiterate female family providers. Recife, PE, Brazil

Figure 2 shows the thematic map of the spatial distribution of the socioeconomic indicators. Intra-urban disparities are observed in the city in terms of the illiteracy rate (Figure 2A), homes in conditions of poverty (Figure 2B), proportion of pregnant women without prenatal care (Figure 2C), and illiterate female family providers (figure 2D).

## Discussion

The HIV detection rate in pregnant women in the city of Recife, corresponding to 3.55 cases/1,000 live births during the study period, surpasses the Brazilian average of 2.0 cases/1,000 live births. This rate was three times higher than the regional average in the Northeast, corresponding to 1.1 case/1,000 live births. This fact demands even more efficient strategies to achieve the program targets^4^.

This high incidence rate reaffirms the understanding of the Aids epidemic in Brazil as a multifaceted phenomenon that comprises a combination of regional sub-epidemics of different magnitudes, dynamics and populations at risk, which are the most affected in each location^6^.

Despite this panorama, one of the challenges that need to be overcome in coping with HIV in pregnant women is the expansion of the coverage rate of prenatal consultations. As an aggravating factor, there is the difficulty of health services to incorporate recommendations for anti-HIV serology screening as a routine test. Although available in the cities, either the professionals do not request the tests or the women do not take it^11^^-^ 12.

The absence, late onset or low quality of prenatal care are appointed as risk factors for maternal-infant HIV transmission, as it induces a late maternal diagnosis and compromises the establishment of the other prophylaxis steps in due time, reducing the efficacy of preventive actions^6^^-^ 7 ^,^ 13 ^-^ 14. Thus, guaranteed care quality and strict monitoring during prenatal care guarantee the early detection of illnesses and minimize maternal-infant health damage.

As observed, the dissemination of the infection among the pregnant women was not random, as it showed a cluster pattern according to the Moran Index, indicating that neighboring regions show similarities. Clusters were evidenced in the neighborhoods Recife, São José and Guabiraba, considered socially underprivileged in studies that support distinctions in these regions, measured by the indicator Living conditions, revealing them as areas with precarious living conditions, in terms of social, economic and infrastructural aspects^15^^-^ 16.

These aspects were similar to the spatial profile of the socioeconomic indicators found in this study. The neighborhoods Recife, São José and Guabiraba lead alarming rates of illiteracy, homes in poverty conditions, proportions of pregnant women who did not get prenatal care and illiterate women as family providers. Thus, when correlating the spatial distribution of HIV in pregnant women with the socioeconomic characteristics of the organization process of the city's urban space, it can be affirmed that neighborhoods distal from the Northwest and Center, with worse socioeconomic and infrastructural conditions, coincided with significantly above average infection rates. The neighborhoods with better indicators showed lower coefficients in the study population.

In the course of the last decade, the country displayed a considerable increase in the urbanization rate, leading to changes in the demographic and epidemiological patterns. Although improvements have been made, such as the reduction of mortality rates due to many diseases and increased life expectancy, the persistence of diseases associated with precarious living conditions still represents a challenges, demanding surveillance models that involve the social context^17^.

The overlaps in spatial events permit associating the maternal-infant transmission of HIV with social vulnerability, in view of the socioterritorial inequalities and health inequities, to explain the high case numbers found in these areas.

Similarly to this study, in Vitória, ES, a greater concentration of cases was found in neighborhoods with a lower urban quality, indicating a pauperization process of Aids and the need to prioritize these locations in actions to reduce the vertical transmission rates^14^.

The influence of social and gender inequalities in the incidence of HIV also finds support in other studies on the spatial distribution of Aids conducted in different contexts^1^^-^ 3 ^,^ 6. As opposed to what was found at the start of the epidemic, when the data proved the concentration of cases among individuals of higher purchasing power and education level, currently, the expansion of the disease progressively affects more vulnerable, poor and marginalized populations.

In addition, Aids is again concentrating in large urban centers, characterized by socioeconomic disparities. This upsurge hampers structural actions aimed at social insertion, which are considered effective to fight the disease^18^.

Concerning the social aspect, public health organizations acknowledge Aids as a disease that derives from inequalities and not from poverty itself, considering that gender, income and regional differences, health service access, sexual and racial orientation aggravate the vulnerability conditions of poor populations in terms of prevention, treatment and effectiveness of the Brazilian response to the disease^19^. In addition, the dissemination of the epidemic and its impacts differ among populations, making it fundamental to recognize the particularities of this process during the elaboration and execution of policies and program strategies aimed at attending to groups vulnerable to exposure to HIV^20^.

The neighborhoods Recife and São José, identified as high-risk areas for the vertical transmission of HIV, are neighbors and located in the central and most urban part of the city. The neighborhood Recife is a touristic harbor area and trading post^15^.

Guabiraba, located in the Northwest of the city, has the city of Olinda as one of its geographic limits, which similarly receives thousands of people in the Carnival period. The socioeconomic standard in Guabiraba is lower to the average in the city, housing a very young population, with higher rates of illiteracy and homes in poor conditions^21^.

Due to traditional cultural touristic programs like Carnival, these neighbors attract visitors from different origins. Thus, they cause an intense flow of people and a high floating population density, aspects that make preventive actions against HIV infection more difficult^22^ and can also contribute to the existence of case clusters. These factors exacerbate the female vulnerability to HIV and demand continuing measures to encourage safe sexual behavior, surpassing the periods of festive events, mainly in locations where many people circulate.

The existence of spatial self-correlation of HIV detection rates in pregnant women demonstrates that neighboring areas tend to present more similar rates as, differently from what happens among distant regions, they tend to share the same characteristics, thus favoring the development of clusters. Case clusters do not only result from people's chosen approximation, but are mainly the product of a series of urban and political factors that favor the spatial segregation^23^.

In addition, an area classified in the epidemiological transition phase was verified^9^, as it contains areas with opposite neighboring rates, showing high and low HIV detection rates in pregnant women. In these areas, actions to control maternal infection and prevent vertical transmission should be intensified, with a view to reducing the avoidable cases of seropositive children.

When the spatial distribution of the socioeconomic indicators is combined with the locations showing the highest case incidence rates, it is confirmed that the clusters coincide with areas in precarious conditions. Therefore, the organization of the space takes the form of different illness risks for the population due to HIV.

As affirmed knowing the spatial distribution of the pregnant women infected by HIV in the community is fundamental to estimate the risk of vertical transmission, deepen the analysis between environment and the development of infections and guide preventive measures^14^.

The main limitation in this study referred to the use of secondary data, with possible influence from factors like underreporting, duplicated records and problems to complete the information. Nevertheless, the choice of this type of source did not make the analyses impossible, nor the confrontation of information.

## Conclusion

Spatial dependence was identified in the geographic distribution of the HIV detection rate in pregnant women, consequently reflecting the spatial self-correlation of the vertical transmission. This indicates a high probability of similar illness in adjacent areas, according to their relative position in space, where the living conditions and infection rates in neighboring areas influence the occurrence of HIV in a certain neighborhood.

Clusters were identified in the neighborhoods Recife and São José, located in the Center, and in Guabiraba, a neighborhood in the distal Northwestern part of the city, which are considered high-risk areas for infection in pregnant women, due to the similar grouping, with the highest rates.

Illiteracy, absence of prenatal care and poverty were relevant to the risk of vertical HIV transmission, converging to the grouping of cases in disadvantaged regions. Areas with better living conditions concentrated low HIV rates among pregnant women, while higher rates were mainly observed in socially disadvantaged regions, aspects that identify the existence of a socioeconomic gradient in the distribution of the epidemic.

These findings permit adapting interventions to the specific needs of the population, as well as prioritizing resources to the most vulnerable locations for the infection of pregnant women, contributing to reduce health inequities.

It is highlighted that caution is fundamental when interpreting the results found, as no inferences should be made regarding individuals, considering that the fact of belonging to the same region or layers does not expose them to the same risk situation observed for an aggregated individual.
